# Comprehensive mapping infection-enhancing epitopes of dengue pr protein using polyclonal antibody against prM

**DOI:** 10.1007/s00253-015-6538-9

**Published:** 2015-03-31

**Authors:** Yayan Luo, Xiaolan Guo, Huijun Yan, Danyun Fang, Gucheng Zeng, Junmei Zhou, Lifang Jiang

**Affiliations:** 1 Guangzhou Brain Hospital (Guangzhou Huiai hospital, the affiliated hospital of Guangzhou Medical University), Guanghzou, 510370 China; 2 Key Laboratory for Tropic Diseases Control, Ministry of Education of China, Department of Microbiology, Zhongshan School of Medicine, Sun Yat-sen University, Guangzhou, 510080 China

**Keywords:** Dengue virus, pr protein, Epitope, Synthetic peptides, Antibody-dependent enhancement

## Abstract

Dengue vaccine development is considered a global public health priority, but the antibody-dependent enhancement (ADE) issues have critically restricted vaccine development. Recent findings have demonstrated that pre-membrane (prM) protein was involved in dengue virus (DENV) infection enhancement. Although the importance of prM antibodies have been well characterized, only a few epitopes in DENV prM protein have ever been identified. In this study, we screened five potential linear epitopes located at positions pr1 (1-16aa), pr3 (13-28aa), pr4 (19-34aa), pr9 (49-64aa), and pr10 (55-70aa) in pr protein using peptide scanning and comprehensive bioinformatics analysis. Then, we found that only pr4 (19-34aa) could elicit high-titer antibodies in Balb/c mice, and this epitope could react with sera from DENV2-infected patients, suggesting that specific antibodies against epitope peptide pr4 were elicited in both DENV-infected mice and human. In addition, our data demonstrated that anti-pr4 sera showed limited neutralizing activity but significant ADE activity toward standard DENV serotypes and imDENV. Hence, it seems responsible to hypothesize that anti-pr4 serum was infection-enhancing antibody and pr4 was infection-enhancing epitope. In conclusion, we characterized a novel infection-enhancing epitope on dengue pr protein, a finding that may provide new insight into the pathogenesis of DENV infection and contribute to dengue vaccine design.

## Introduction

Dengue virus (DENV1-4) causes the most significant arboviral disease in the tropical and subtropical areas of the world, with an estimated 390 million infections per year (Guzman and Kouri 2002). While most DENV infections are asymptomatic or lead to self-limited dengue fever (DF), a growing number of patients present potentially fatal clinical manifestations including dengue hemorrhagic fever (DHF) and dengue shock syndrome (DSS) (Bhatt et al. 2013). Although infection with DENV is a growing global public health concern and imposes one of the largest socioeconomic burdens in the world, approved vaccine remains unavailable despite decades of effort (Coller and Clements 2011; Miller 2010).

The immunopathogenesis of DENV infection is still poorly understood. It is well established that the development of DHF/DSS is associated with sequential infection with different serotypes (Gubler 2006). Also, primary infection of infants born to dengue-immune mothers may be at greater risk of developing severe disease (Simmons et al. 2007). The hypothesis of antibody-dependent enhancement (ADE) of infection has been postulated because of these observations. It is reasoned that subneutralizing antibodies promoted virus uptake into Fc receptor-bearing cells, leading to increased DENV-infected cells and eventually a higher virus load (Halstead 1970; Halstead and O’Rourke 1977; Halstead 2003). Hence, a successful and safe vaccine must be tetravalent, capable of providing long-term protection against all four serotypes simultaneously (Guy et al. 2011).

Dengue is a single-stranded, positive-sense, RNA virus with a genome of approximately 11 kb. The virion contains three structural (capsid (C), pre-membrane (prM), and envelope (E)) and seven nonstructural (NS) proteins (NS1, NS2a, NS2b, NS3, NS4a, NS4b, and NS5). prM is a 166-amino-acid protein and acts as a chaperone for correct folding of the E protein during virus assembly and maturation (Li et al. 2008; Mukhopadhyay et al. 2005). prM contains a furin cleavage site and is cleaved by the host cell endoprotease furin into a C-terminal M protein and an N-terminal 91-amino-acid precursor peptide (pr protein), resulting in the formation of mature infectious virus (Yu et al. 2008). Interestingly, cells infected with DENV secrete a heterogeneous mixture varying from fully mature (containing M), partially mature virions (containing a mixture of prM and M) to fully immature (containing prM) due to inefficient cleavage of prM to M by furin during DENV maturation (Rodenhuis-Zybert et al. 2008; Cherrier et al. 2009; Junjhon et al. 2010). It has been proved that fully immature virus particles are inherently noninfectious whereas fully mature and partially mature virus are infectious (Rodenhuis-Zybert et al. 2008, 2010, 2011; Dejnirattisai et al. 2010). Additionally, the enhancement or neutralization effect of antibodies seems to be dependent on the maturation state of virions (Dejnirattisai et al. 2010; Rodenhuis-Zybert et al. 2010; Nelson et al. 2008).

It has been previously reported that anti-prM antibodies are commonly found in sera of patients with DENV infection (Dejnirattisai et al. 2010; Bray and Lai 1991; Cardosa et al. 2002; Se-Thoe et al. 1999). However, the potential role of prM antibodies during DENV infection was long overlooked as numerous functional studies have revealed that fully immature particles are noninfectious (Colpitts et al. 2011). Interestingly, recent studies on mouse and human antibodies showed that prM antibodies render virtually noninfectious immature DENV particles highly infectious (Dejnirattisai et al. 2010; Rodenhuis-Zybert et al. 2010; Chan et al. 2012). In addition, anti-prM antibodies are found to play important roles in patients with both primary infection and second infection (Dejnirattisai et al. 2010; Lai et al. 2008). Furthermore, many studies have suggested that these antibodies against prM do not well neutralize DENV infection but potently promote ADE infection among four DENV serotypes (Dejnirattisai et al. 2010; Beltramello et al. 2010). It has also been suggested that anti-prM antibodies could enhance wild-type DENV infection (Huang et al. 2005, 2006) and increase disease severity in infants upon primary infection (Chau et al. 2009). Most importantly, previous study showed a positive correlation between the circulating prM antibody level and disease severity (Rai et al. 2008). Taken together, these studies suggest that prM-specific monoclonal antibodies (mAbs) have a potential role to enhance DENV infection in humans and thus may contribute to the development of severe disease.

A recent report detecting the acute B cell response in dengue patients has found that DENV infection results in significant B cell activation at days 4–7 after onset of fever (Balakrishnan et al. 2011). prM protein, like E protein, is a major target in the humoral immune response to DENV (Cardosa et al. 2002; Beltramello et al. 2010). Epitopes are the focus of pathogenesis research as well as the development of vaccine and diagnostic reagent (Wu et al. 2001, 2003; Jiang et al. 2010). Epitopes in the E (Li et al. 2012; de Alwis et al. 2012; Lin et al. 2012; Hughes et al. 2012; Wahala et al. 2012), NS1( Wu et al. 2001, 2003; Jiang et al. 2010; Steidel et al. 2012), NS4a, and C (Anandarao et al. 2005) have been well mapped. Although there have been attempts to locate the epitopes of prM (Falconar 1999; Huang et al. 2008; Song et al. 2013; Luo et al. 2013), the functional roles of the prM protein in DENV infection and the precise antigenic structures of prM are not yet fully understood. Thus, in this study, we mapped the potential B cell epitopes in the pr protein of DENV with peptide scanning and comprehensive bioinformatics analysis. We also investigated the neutralizing versus enhancing capacity of antisera of epitope peptide pr4 toward standard DENV1-4 particles and imDENV particles.

## Materials and methods

### Cells and viruses

C6/36 cells, baby hamster kidney-21 (BHK-21) cells, human adenocarcinoma LoVo cells, and human erythroleukemic K562 cells were maintained as previously described (Luo et al. 2013). All cells were purchased from ATCC.

DENV1 strain Hawaii (GenBank EU848545), DENV2 strain New Guinea C (NGC) (GenBank AF038403), DENV3 strain H87 (GenBank M93130), DENV4 strain H241 (GenBank AY947539), and JEV (GenBank AF315119) were propagated on C6/36 cells, while fully imDEVN2 NGC strain was produced on furin-deficient LoVo cells as described before (Rodenhuis-Zybert et al. 2008; Luo et al. 2013). The infectious titers of virus particles were detected by plaque assay on BHK-21 cells and viral RNA levels were measured by real-time quantitative RT-PCR (qRT-PCR) (Luo et al. 2012). All viruses were kindly provided by Chinese Center for Disease Control and Prevention, Beijing, China. All viruses were deposited in GenBank.

### Antibodies and human serum samples

2H2 (IgG2a anti-DENV1-4 prM) and 4G2 (IgG2a anti-all flavivirus E) hybridomas were purchased from ATCC.

To generate polyclonal antibody (pAb) against prM, the DENV-2 prM protein was expressed in *E. coli* and then purified by electroelution. The purified protein was used to immunize adult male New Zealand rabbits to produce pAb directed to prM. Anti-prM pAb was purified by caprylic acid–ammonium sulfate precipitation and protein A affinity column (GenScript, USA); then, the specificity and titer of the antibody were verified by Western blot and enzyme-linked immunosorbent assay (ELISA) (Feng et al. 2013).

Human serum samples were obtained and identified as described before (Luo et al. 2013). The use of human sera was approved by the ethical committee of Haizhu District Center for Disease Control and Prevention of Guangzhou, China. The study was also approved by the Ethics Committee of Sun Yat-sen University.

### Peptide synthesis and epitope mapping using anti-prM pAb

Fourteen overlapping peptides (each with 16 amino acids, overlap of 10) covering the pr protein were designed and synthesized (purity >95 %, China Peptides Co., Ltd) (Table 1). Then, the predominant linear B epitopes on pr protein were mapped by ELISA using anti-prM pAb and 14 overlapping peptides.

**Table 1:** A total of 14 overlapping peptides of pr protein (each with 16 amino acids, overlap of 10)

Peptide	Peptide sequence and location	Peptide	Peptide sequence and location
pr1	^1^fhlttrngephmivsr^16^	pr8	^43^elcedtityncpllrq^58^
pr2	^7^ngephmivsrqekgks^22^	pr9	^49^ityncpllrqnepedi^64^
pr3	^13^ivsrqekgksllfkte^28^	pr10	^55^llrqnepedidcwcns^70^
pr4	^19^kgksllfktengvnmc^34^	pr11	^61^pedidcwcnststwvt^76^
pr5	^25^fktengvnmctlmamd^40^	pr12	_67_wcnststwvtygtcta^82^
pr6	^31^vnmctlmamdlgelce^46^	pr13	^73^twvtygtctatgehrr^88^
pr7	^37^mamdlgelcedtityn^52^	pr14	^79^tctatgehrrekr^91^

### ELISA

To screen specific epitopes that directly bind with the pr protein, ELISA was performed with anti-prM pAb. The ELISA plate was coated with 100 μl synthetic peptides at a concentration of 10 μg/ml at 4 °C overnight and blocked at 37 °C for 2 h. After washing three times with PBS with 0.1 % Tween-20 (PBST), the plates were incubated with 100 μl anti-prM pAb diluted in 1:200 at 37 °C for 1 h. Bound antibodies were detected with 1:5000 diluted HRP-conjugated goat-anti-rabbit IgG at 37 °C for 45 min. Then, plates were washed three times and incubated with TMB substrate. The reaction was stopped with 2 N H_2_SO4, and the plates were measured at 450 nm using a microplate autoreader.

### Bioinformatics analysis of DENV2 pr B cell epitopes

The potential linear epitope on pr protein was predicted using DNASTAR software and ExPaSy multiple bioinformation software as described previously (Luo et al. 2013).

### Competitive-inhibition assay

The ELISA plate was coated with anti-prM pAb. Next, synthetic peptide was added 0.1 μg per well and purified prM protein was added simultaneously. Then, the same procedures as described in “ELISA” were followed.

### Immunization assay and protection assay in adult Balb/c mice

All procedures involving animals were approved by the Animal Experimentation Ethics Committee of Sun Yat-sen University and carried out by a licensed individual with an ethical approval number of 2012/0081.

Considering the results of peptide scanning and bioinformatics analysis together, we screened five predominant linear B epitopes located at regions pr1 (1-16aa), pr3 (13-28aa), pr4 (19-34aa), pr9 (49-64aa), and pr10 (55-70aa). Then, we chose epitope peptides (pr1, pr3, pr4, pr9, and pr10) and control peptide PM10 (SQNPPHRHQS) to immunize Balb/C mice and collected anisera for further study as described previously (Luo et al. 2013). The antibody titer of mice sera was detected by using ELISA.

The protection experiment was performed as described previously (Luo et al. 2013). The viral RNA levels of serum samples were detected using qRT-PCR (Luo et al. 2012). All animals were euthanized by using carbon dioxide (CO_2_) according to NC3Rs standard procedures, and the experiment was terminated.

### Plaque reduction neutralization test (PRNT) and antibody-dependent infection enhancement (ADE) assay

To determine the ability of prM-specific antibodies to neutralize or enhance DENV infection in vitro, PRNT and ADE assay were carried out on BHK-21 cells and K562 cells respectively as described in our previous study (Luo et al. 2013).

### Flow cytometry

The infected K562 cells were fixed, permeabilized, and stained with 4G2 conjugated to Alexa-Fluor-488 (Invitrogen). The percent of infected cells was determined by flow cytometry.

### Statistical analysis

Statistical analyses were performed in GraphPad Prism5.0 software. ANOVA Tukey’s posthoc statistical tests were used for pairwise comparisons of multiple groups. A *p* value of less than 0.05 was considered statistically significant.

## Results

### Mapping of the antigenic epitopes on the pr protein using anti-prM pAb

**Fig. 1:**
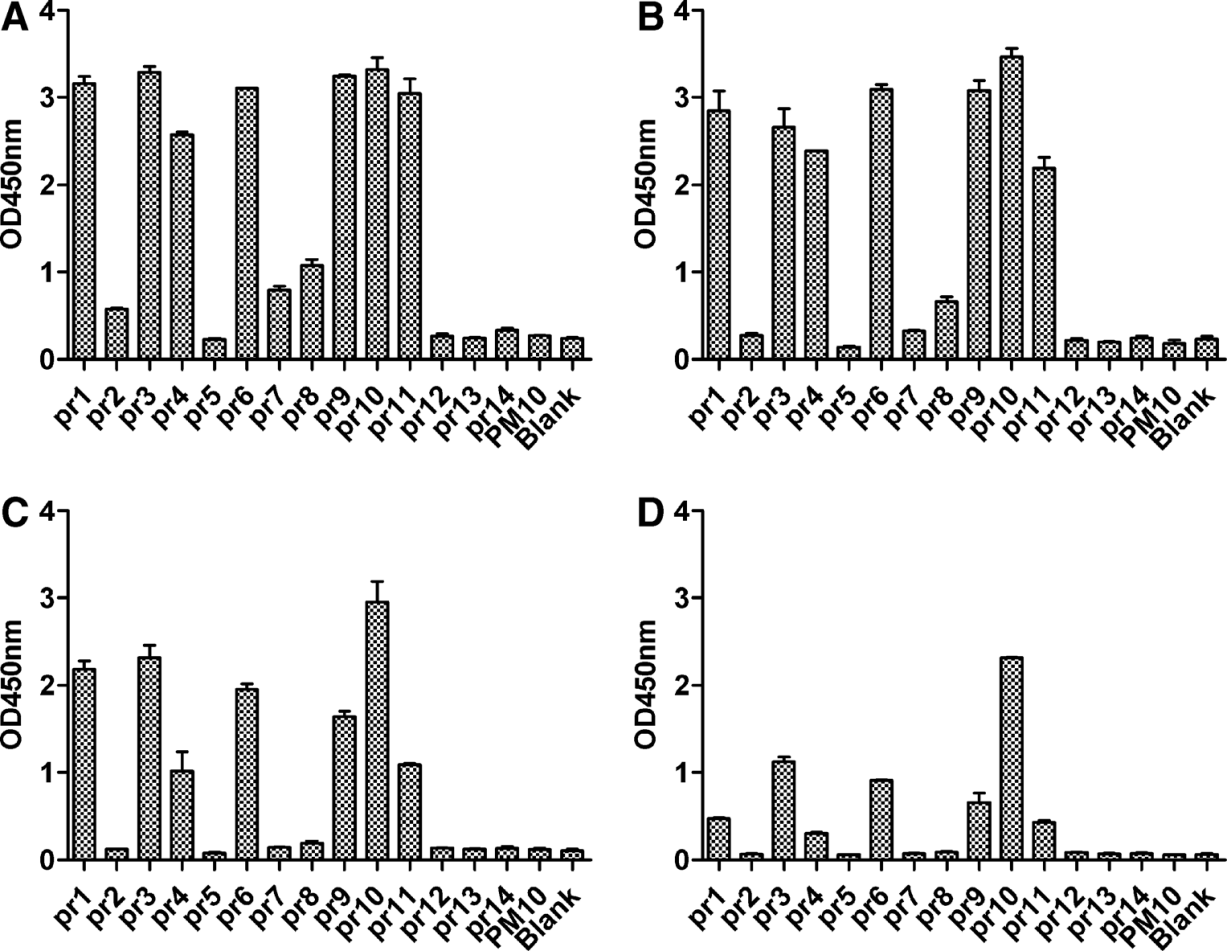
Comprehensive identification of linear B cell epitopes in DENV2 pr protein using anti-prM pAb by ELISA. Seven potential B cell epitopes in pr protein were mapped. prM pAb was diluted 200-fold (**a**), 1000-fold (**b**), 5000-fold (**c**), and 10,000-fold (**d**). PM10 was used control peptide. Data are expressed as means of at least three independent experiments. The *error bars* represent standard deviations (SD). If there is no error bar, it is not that no variations among three independent experiments but that the variations are too small to show in the figure

**Fig. 2:**
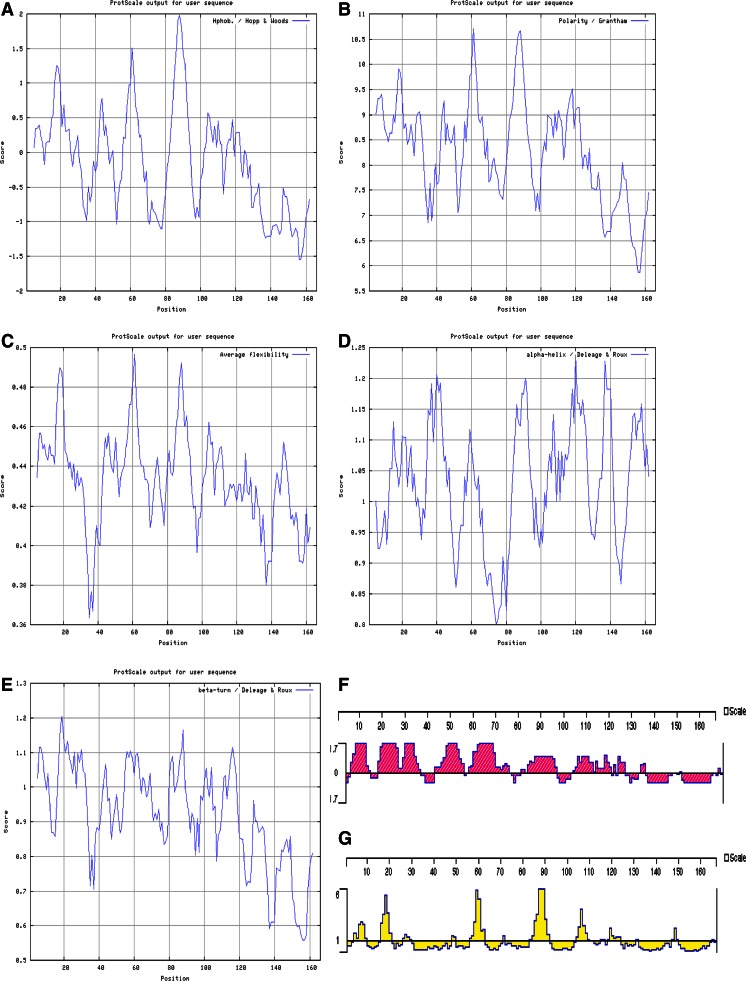
General evaluation of DENV2-pr epitopes with bioinformation software. **a** Hopp and Wood hydrophilicity, **b** Granthan polarity, **c** Bhaskaran and Ponnuswamy flexibility, **d** Deleage and Roux alpha-helix regions, **e** Deleage and Roux beta-turn regions, **f** Jameson and Wolf antigenicity, and **g** Emini accessibility

To identify the potential antigenic epitopes on the pr protein, 11 partially overlapping peptides (pr1–pr14) were screened by ELISA using anti-prM pAb at various dilutions. The results showed that seven peptides (pr1, pr3, pr4, pr6, pr9, pr10, and pr11) were recognized by anti-prM pAb (Fig. 1), suggesting that these peptides may be the potential B cell epitopes in pr protein. All the other seven overlapping peptides and control peptide PM10 failed to bind with pAb against prM. Our previous study (Luo et al. 2013) has reported that the potential linear epitopes on pr protein predicted using DNASTAR software and ExPaSy multiple bioinformation software were located at regions 3-12aa, 12-26aa, 55-66aa, and 82-91aa (Fig. 2). Considering the results of peptide scanning and bioinformatics analysis together, five predominant epitope peptides (pr1, pr3, pr4, pr9, and pr10) were chosen to immunize Balb/C mice for further study.

### Immunogenicity of synthetic peptides on Balb/c mice

**Fig. 3:**
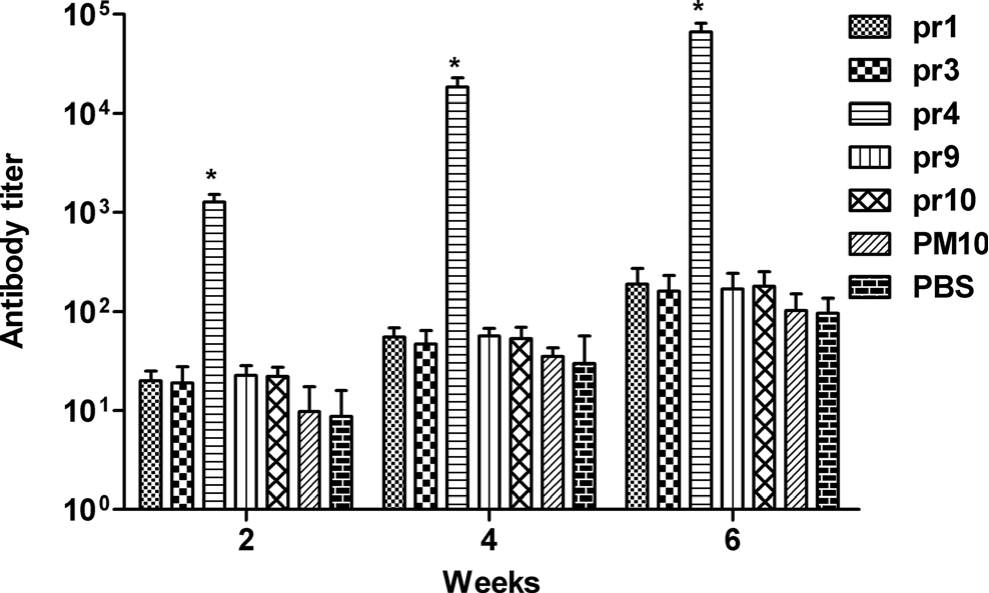
Serum antibody titers induced in Balb/C mice immunized with the synthetic peptides. Seven groups (pr1, pr3, pr4, pr9, pr10, PM10, and PBS), ten mice for every group, were immunized three times with 100 μg of immunogens. All peptides were coupled to KLH before immunization. PM10 and PBS were used as controls. The antisera were collected on week 2, 4 and 6 via tail vein, and the antibody titer of mice sera was detected by ELISA. The antibody titer of anti-pr4 sera was remarkably higher than that of the antisera from groups of pr1, pr3, pr9, pr10, PM10, and PBS at any time point. Data are expressed as means of at least three independent experiments. The *error bars* represent standard deviations (SD); *p < 0.05 vs other groups (pr1, pr3, pr9, pr10, PM10, and PBS)

According to ELISA results, high titer of antibodies was only found in mice immunized with pr4. The antibody titer of anti-pr4 sera was remarkably higher than that of the antisera from groups of pr1, pr3, pr9, pr10, PM10, and PBS at any time point (*p* < 0.05). Furthermore, antibody titers of pr4 increased progressively with each sequential immunization. Thus, immunization with pr4 elicits high-titer antibodies in Balb/c mice (Fig. 3).

### Protection response in adult Balb/c mice

**Fig. 4:**
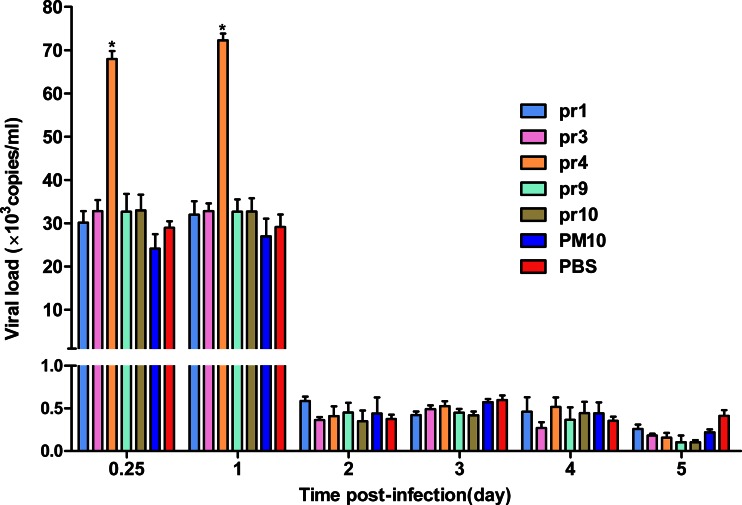
Viral RNA copy numbers in immunized mice after infected with DENV2 NGC strain. Two weeks after the last immunization, mice were inoculated intraperitoneally with 10^6^ PFU DENV2. Viral RNA copy numbers of sera were measured at days 0.25, 1, 2, 3, 4, and 5 post-infection by qRT-PCR. PM10 and PBS were used as controls. The viral RNA copy numbers in the pr4 group were significantly higher than that in the other groups (pr1, pr3, pr6, pr9, PM10, and PBS) at days 0.25 and 1 post-infection. Data are expressed as means of at least three independent experiments. The *error bars* represent standard deviations (SD); *p < 0.05 vs other groups (pr1, pr3, pr9, pr10, PM10, and PBS)

As shown in Fig. 4, high levels of viral RNA in all groups were observed at days 0.25 and 1 post-infection, and the viral RNA levels in the pr4 group were significantly higher than that in the other groups (pr1, pr3, pr6, pr9, PM10, and PBS) at days 0.25 and 1 post-infection (*p* < 0.05). However, all groups showed a rapidly decreased viremia levels at days 2, 3, 4, and 5 post-infection.

### Characterization of epitope peptide pr4

To analyze the conservation of epitope peptide pr4 (19-34aa), we performed a sequence alignment of the pr protein sequences of DENV1-4, YFV, WNV, JEV, and TBEV using DNASTAR software. Alignment results showed that pr4 epitope sequence was highly conserved among four DENV serotypes but not among other flaviviruses (Table 2).

**Table 2:** Sequence alignment of amino acid residues 19 to 34 of the pr proteins of flaviviruses

Virus^a^	Amino acid sequence
DENV1	RGKSLLFKTAAGVNMC
DENV2	KGKSLLFKTEDGVNMC
DENV3	RGKSLLFKTASGINMC
DENV4	RGRPLLFKTTEGINKC
WNV	VTDVITIPTAAGKNLC
JEV	IADVIVIPTSKGENRC
YFV	LGKTFSVGTGNCTTNI
TBEV	TQVRVENGTCVILATD

^a^The protein sequences of DENV1, DENV2, DENV3, DENV4, WNV, JEV, YFV, and TBEV were retrieved from GenBank with accession numbers EU848545, AF038403, M93130, AY947539, DQ211652, AF315119, X03700, and AY182009, respectively

**Fig. 5:**
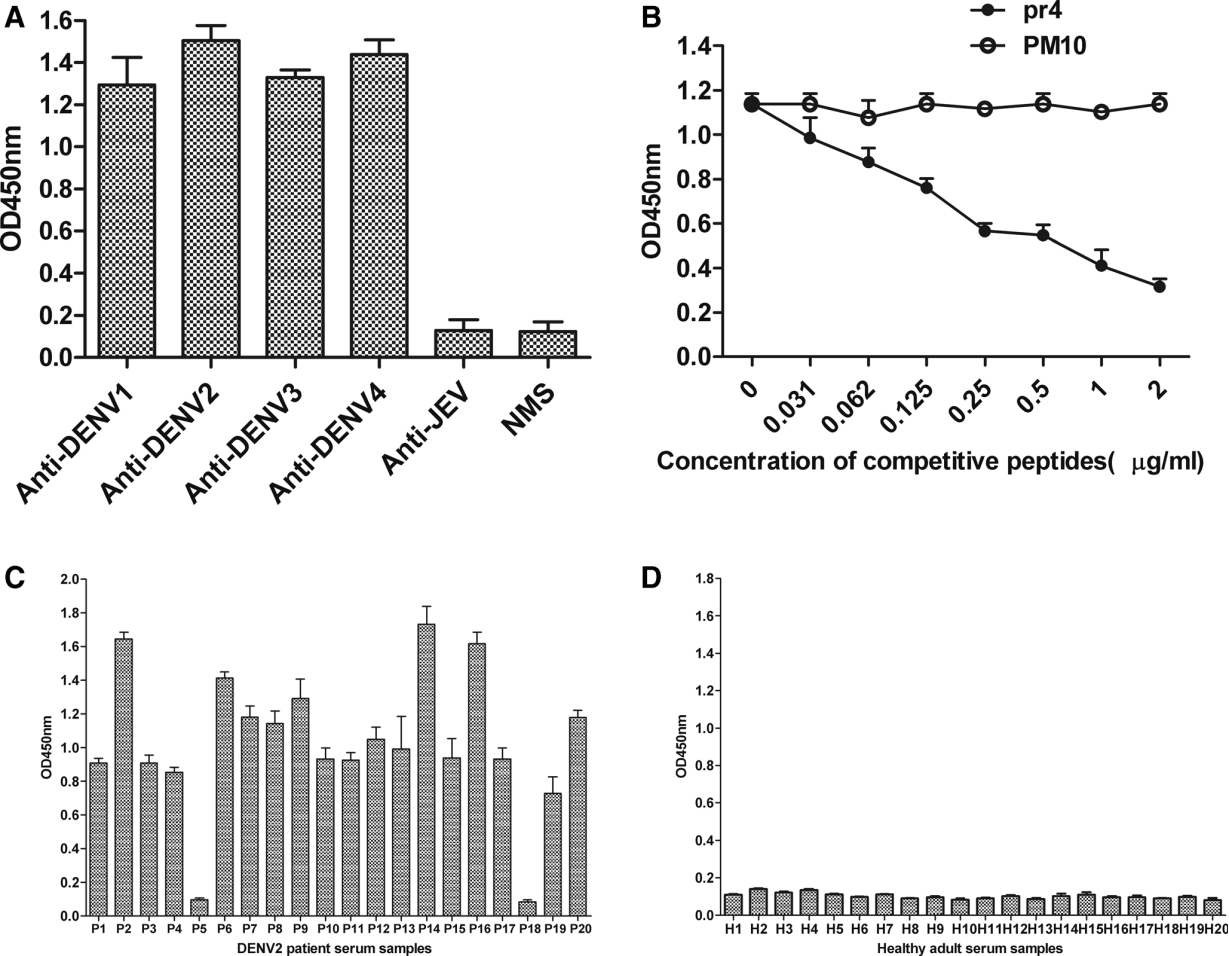
Characterization of epitope peptide pr4. **a** ELISA reactivities of synthetic peptide pr4 with immunized mice sera. Synthetic peptide pr4 could react with anti-DENV1-4 mice sera but not with anti-JEV mice sera and normal mice sera (NMS). **b** Competitive inhibition of prM protein binding to anti-prM pAb by synthetic peptide pr4. Competitive ELISA was performed using pr4 as competitor of prM protein. The reaction activity of anti-prM pAb with prM protein was inhibited markedly by pr4 in a concentration-dependent manner. **c**, **d** ELISA reactivities of synthetic peptide pr4 with sera from 20 DENV2-infected patients (**c**) and 20 healthy adults (**d**). pr4 was able to react with sera from 18 patients infected with DENV2 but not with sera from healthy adults. PM10 was used as control. Data are expressed as means of at least three independent experiments. The *error bars* represent standard deviations (SD). If there is no error bar, it is not that no variations among three independent experiments but that the variations are too small to show in the figure

We next evaluated whether the synthetic peptide pr4 could be recognized by anti-DENV1-4 mice sera. Synthetic peptide pr4 could react with anti-DENV1-4 mice sera but not with anti-JEV mice sera and normal mice sera (NMS) (Fig. 5a), suggesting that synthetic peptide pr4 is a DENV serocomplex cross-reactive epitope-based peptide.

To prove further that synthetic peptide pr4 was the epitope of pr protein, a peptide competitive inhibition assay was performed to determine whether the pr4 peptide competed with prM protein for reactivity with anti-prM pAb. The reaction activity of anti-prM pAb with prM protein was inhibited markedly by pr4 in a concentration-dependent manner (Fig. 5b).

Then, we confirm the reactivity of synthetic peptide pr4 with 20 DENV2 patient serum samples. As shown in Fig. 5c, d, pr4 was able to react with sera from 18 patients infected with DENV2. The sensitivity of pr4 serologic test was 90 %. In addition, patients showed difference in the level of antibody. However, all of the sera from 20 healthy adults failed to bind with pr4. These results demonstrated that specific antibody response against pr4 epitope was elicited during natural DENV infection.

### Neutralizing activities of anti-pr4 sera

**Fig. 6:**
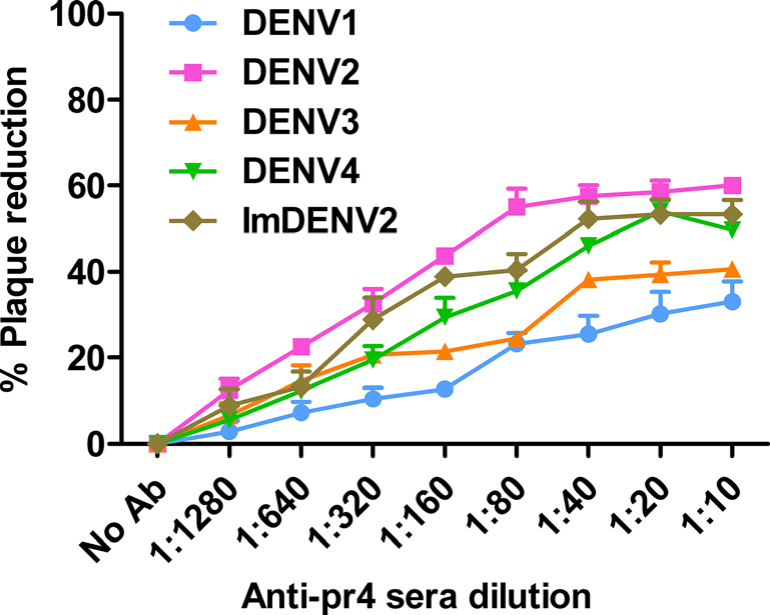
Neutralizing activities of anti-pr4 sera toward standard DENV1-4 and imDENV2. Fifty PFU viruses were mixed with 2-fold serially diluted antibody for 1 h at 37 °C. Neutralizing activities were detected by plaque reduction assay using BHK-21 cells. Anti-pr4 sera show poor neutralizing activities with standard DENV1-4 and imDENV2. Data are expressed as means of at least three independent experiments. The *error bars* represent standard deviations (SD). If there is no error bar, it is not that no variations among three independent experiments but that the variations are too small to show in the figure

Plaque reduction neutralization test results showed that anti-pr4 sera failed to completely neutralize infection with the neutralization level varying from 33 to 60 %, and the partial neutralization was cross-reactive among four DENV serotypes (Fig. 6). Anti-pr4 sera, like many other prM antibodies, did not exhibited high neutralizing activity. Also, virtually noninfectious imDENV was neutralized by anti-pr4 sera and the titration curve for standard DENV2 and imDENV2 were similar (Fig. 6). These results suggest that anti-pr4 sera exhibited weak neutralizing activities against standard DENV-4 and imDENV2.

### ADE activities of anti-pr4 sera

**Fig. 7:**
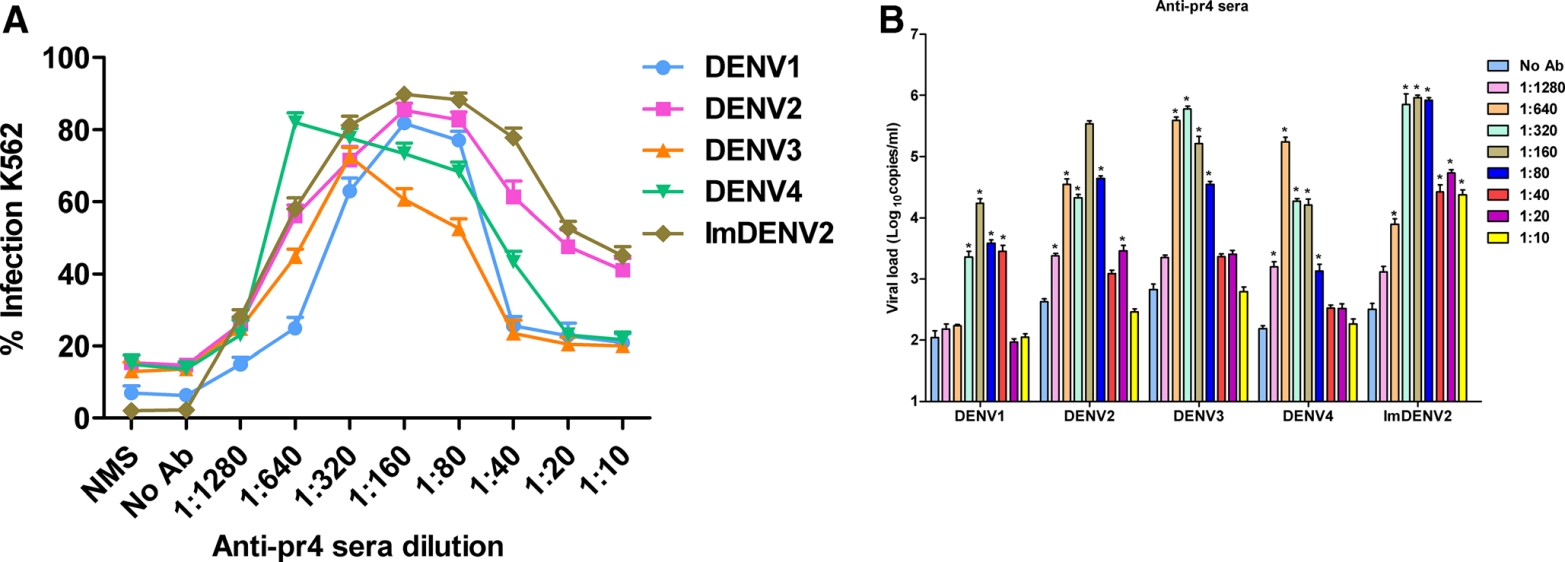
ADE of DENV infection in K562 cells mediated by anti-pr4 sera. Twofold serially diluted antibodies and an equal volume of DENV (MOI of 1) were mixed for 1 h at 37 °C and added to K562 cells. **a** The percent of infected K562 cells was measured at 3 days post-infection by flow cytometry. **b** Viral RNA levels of supernatants were accessed at 4 days post-infection by qRT-PCR. Anti-pr4 sera showed significant ADE activities toward standard DENV and imDENV2. NMS was used as control. Data are expressed as means of at least three independent experiments. The *error bars* represent standard deviations (SD). If there is no error bar, it is not that no variations among three independent experiments but that the variations are too small to show in the figure. *p < 0.05 vs No Ab

The flow cytometry results indicated that anti-pr4 sera was able to enhance infection varying from 6.3 to 87.6 % over a wide range of antibody concentration among four DENV serotypes (Fig. 7a). Then, we detected enhancement of imDENV2 infection using a constant amount of virus particles identical to standard DENV2. We found that infection of virtually noninfectious imDENV was also significantly enhanced by anti-pr4 sera. To further confirm the enhancing capacity of anti-pr4 sera, we evaluated viral RNA copies in infected supernatants using qRT-PCR (Fig. 7b). In agreement with flow cytometry results, anti-pr4 sera caused a significant increase of viral load over a large antibody concentration range (*p* < 0.05). In summary, these results suggest that anti-pr4 sera showed significant ADE activities toward standard DENV and imDENV2.

## Discussion

Recent findings demonstrated that anti-prM antibodies play critical roles in human immune responses to DENV. These antibodies showed limited neutralizing activity but significant ADE activity (Dejnirattisai et al. 2010; Rodenhuis-Zybert et al. 2010, 2011; Chan et al. 2012; Lai et al. 2008; Beltramello et al. 2010; Huang et al. 2006). Although the importance of prM antibodies has been well reported, the precise antigenic structures and the functional roles of the prM protein remain poorly understood. Thus, in this study, we identified the potential B cell epitopes in the dengue pr protein and investigated the ability of antibody against epitope peptide pr4 to enhance infection of standard DENV1-4 and imDENV2.

In the present study, by using peptide scanning and bioinformatics analysis, we mapped five potential linear B cell epitopes (pr1, pr3, pr4, pr9, and pr10) in the dengue pr protein. However, immunization assay results showed that only epitope peptide pr4 was capable of inducing strong humoral immune response in mice and showed to be highly immunogenic. Sequence alignment results demonstrated that pr4 is a DENV serocomplex cross-reactive epitope peptide. In our experiments, epitope peptide pr4 showed strong reactivity with mouse antisera against DENV1-4 but not with anti-JEV mice sera. Previous studies have reported that anti-prM antibodies could distinguish infection with DENV from infection with JEV (Cardosa et al. 2002; Hua et al. 2010). To our knowledge, cross-reactivity among flaviviruses has been a great diagnostic obstacle, especially for members of DENV. Hence, epitope peptide pr4 could be potentially applied in serological monitoring and differential diagnosis of DENV infection. Most, importantly, human sera from patients infected with DENV2 also specifically recognized pr4, suggesting that epitope peptide pr4 may be valuable for the further development of a DENV-specific diagnostic reagent. Together, these results showed specific antibodies against epitope peptide pr4 were elicited in both DENV-infected mice and human, indicating the importance of pr4 epitope during natural DENV infection.

Our data in this study showed that other four synthetic peptides (pr1, pr3, pr6, and pr9) could not induce high antibody titer in mice. Synthetic peptides representing specific regions of proteins can induce humoral immune responses (Vázquez et al. 2002). It has been reported that short peptide may not exactly imitate the structural of homologous sequence in the native protein (Jemmerson and Hutchinson 1990), because other factors such as neighboring residues and H bonds (within protein) have also influenced the structure obtained by short stretches in whole protein. Most importantly, peptides must contain potential antigenic sites to elicit B cell interaction.

It has been well demonstrated that the anti-prM antibody mediated DENV infection enhancement and play critical roles in DENV pathogenesis (Rodenhuis-Zybert et al. 2008, 2010; Dejnirattisai et al. 2010; Lai et al. 2008; Beltramello et al. 2010; Huang et al. 2006, 2008; Luo et al. 2013). In agreement with these studies, antibody against epitope peptide pr4 described in this study exhibited broad cross-reactivity and poor neutralizing activity but potent ADE activity toward the four standard DENV serotypes and imDENV. In addition, we found that the epitope peptide pr4 indeed induce enhancing antibodies and lead to an increase of viral load in mice sera during protection assay in vivo. Thus, we concluded that anti-pr4 serum was infection-enhancing antibody and pr4 was infection-enhancing epitope. Taken together, we further confirmed prM antibodies could enhance infectivity of DENV and demonstrated the importance of prM antibodies during DENV infection. To date, the molecular mechanism of neutralization and ADE is still not well described. It has been demonstrated that antibodies against prM can enhance infectivity of prM-containing DENV particles by facilitating efficient binding and cell entry of virus-antibody complexes into Fc receptor-bearing cells (Dejnirattisai et al. 2010; Rodenhuis-Zybert et al. 2010).

Anti-pr4 sera showed different neutralizing and ADE activities against different DENV serotypes, suggesting the structural differences in this region that perhaps involve the polarities and side chain orientations of structurally neighboring amino acids. In addition, the well-exposed epitopes available for antibody recognition in the structural change process are likely to vary in different viruses (Nelson et al. 2008; Roehrig 2003). Also, it has been demonstrated that differences in DENV serotype or even strain might be a factor in generation of varying degrees of ADE and dengue severity (Endy et al. 2004; Vaughn et al. 2000; Goncalvez et al. 2007). The differences in ADE between serotypes have become the main obstacle to study immunopathogenesis of DENV infection and develop dengue vaccines. Dejnirattisai et al. (2010) and Beltramello et al. (2010) also have found that four DENV serotypes showed different degrees of ADE.

Although dengue vaccine development is considered a global public health priority, approved vaccines remain unavailable due to the cross-reactive among four DENV serotypes and ADE phenomenon. As reported in the present study and recent reports, prM-specific antibodies are capable of restoring and enhancing the infectivity of prM-containing immature and partially mature (Rodenhuis-Zybert et al. 2008, 2010; Dejnirattisai et al. 2010; Lai et al. 2008; Beltramello et al. 2010; Huang et al. 2006, 2008; Luo et al. 2013). Hence, a better vaccine design approach that minimizes the ADE responses elicited by enhancing epitopes in prM protein may contribute to the success of dengue vaccines. Consequently, it may be important to map various kinds of prM epitopes, which are responsible for enhancing antibodies or neutralizing antibodies. However, only a few epitopes in DENV prM protein have been well characterized (Falconar 1999; Huang et al. 2008; Song et al. 2013; Luo et al. 2013). In this study, we map a novel infection-enhancing epitope on dengue pr protein, a finding that may provide new insight into the pathogenesis of DENV infection and contribute to dengue vaccine design.

In conclusion, we screened a DENV serocomplex cross-reactive epitope pr4 (19-34aa) on pr protein using peptide scanning and comprehensive bioinformatics analysis, and found that this epitope was infection-enhancing. These findings may help to understand pathogenesis of DENV infection and advance the development of dengue vaccine.
